# Comparative studies of macrophage-biased responses in mice to infection with *Toxoplasma gondii* ToxoDB #9 strains of different virulence isolated from China

**DOI:** 10.1186/1756-3305-6-308

**Published:** 2013-10-26

**Authors:** Ai-Mei Zhang, Qian Shen, Min Li, Xiu-Cai Xu, He Chen, Yi-Hong Cai, Qing-Li Luo, De-Yong Chu, Li Yu, Jian Du, Zhao-Rong Lun, Yong Wang, Quan Sha, Ji-Long Shen

**Affiliations:** 1 Anhui Provincial Laboratories of Pathogen Biology and Zoonoses, Department of Microbiology and Parasitology, Anhui Medical University, Hefei, Anhui, China; 2 Central Laboratory of Affiliated Provincial Hospital of Anhui Medical University, Hefei, Anhui, China; 3 Department of lmmunology, Anhui Medical Universty, Hefei, Anhui, China; 4 Department of Biochemistry and Molecular Biology, Anhui Medical University, Hefei, China; 5 Center for Parasitic Organisms, State Key Laboratory of Biocontrol, School of Life Sciences and Key Laboratory of Tropical Diseases and Control of the Ministry of Education, Sun Yat-Sen University, Guangzhou, Guangdong, China; 6 Department of Pathogen Biology, Nanjing Medical University, Nanjing, China

**Keywords:** *Toxoplasma gondii*, Genotyping, Macrophages, Macrophage activation, Signaling pathway

## Abstract

**Background:**

Different from three clonal lineages of *Toxoplasma gondii* in North America and Europe, the genotype China 1 is predominantly prevalent in China. However, there are different virulent isolates within China 1, such as virulent TgCtwh3 and avirulent TgCtwh6, and little is known about differences in macrophage activation between them. The objective of this study focused on cytokine production, phenotype and markers of activated macrophages, and correlated signaling pathway induced by the two isolates.

**Methods:**

Adherent peritoneal macrophages (termed Wh3-Mφ and Wh6-Mφ, respectively) harvested from infected mice were cultured for detection of Nitric Oxide and arginase activity, and activated markers on Wh3-Mφ/Wh6-Mφ were determined by flow cytometry. In *in vitro* experiments, the levels of IL-12p40 and TNF-α were measured using ELISA kits, and mRNA expressions of IL-12p40, TNF-α, iNOS, Arg-1 and Ym1 were assayed by real-time PCR. To confirm the activation state of NF-kB p65 in infected cells stained by IF, protein levels of iNOS, Arg-1, Ym1, nuclear NF-κB p65, and phosphorylation of STAT6/STAT3/IκBα were evaluated by Western Blotting. A one-way ANOVA test was used to compare differences among multiple groups.

**Results:**

The result revealed that contrary to the virulent TgCtwh3, the less virulent TgCtwh6 isolate induced a significant increase in IL-12p40 and TNF-α. Although both isolates down-regulated CD80, CD86 and MHCII molecule expression on macrophages, TgCtwh3 promoted up-regulation of PD-L2 and CD206. Wh6-Mφ generated a high level of NO whereas Wh3-Mφ up-regulated Ym1 and arginase expression at transcriptional and protein levels. In terms of signaling pathway, TgCtwh3 induced phospho-STAT6, conversely, TgCtWh6 led to NF-κB p65 activation.

**Conclusions:**

The virulent TgCtwh3 isolate induced macrophages to polarize toward alternatively activated cells with STAT6 phosphorylation, whereas the less virulent TgCtwh6 elicited the development of classically activated macrophages with nuclear translocation of NF-κB p65. This discrepancy suggests that it is necessary to thoroughly analyze the genotype of TgCtwh3 and TgCtwh6, and to further study other effector molecules that contribute to the macrophage polarization in *T. gondii.*

## Background

*Toxoplasma gondii* is an intracellular parasite capable of infecting a broad spectrum of hosts including up to 30% of the world’s human population [1]. Infection occurs when individuals ingest undercooked meat containing cysts of the parasite or consume food contaminated with oocysts. Although most infections are mild in healthy adults, life-threatening consequences may develop in immunocompromised individuals and *in utero* infection can lead to major defects in the fetus [2].

During acute infection, tachyzoites, the rapidly replicative form of the parasite, elicits an extremely strong type Th1 immune response, characterized by proinflammatory cytokine production such as IL-6, IFN-γ and TNF-α. Macrophages can provide a niche permissive for parasite replication and are the most abundant cell type infected by *Toxoplasma*[3]. It has been clarified that macrophages can be phenotypically polarized by the micro-environment to mount specific functional programs [4]. Polarized macrophages have been classified in two main clades: classically activated macrophages (or M1) whose typical activating stimuli are IFN-γ and LPS, and alternatively activated macrophages (or M2) induced by exposure to IL-4 and IL-13. M1 exhibits antimicrobial functions against intracellular pathogens which is conducted by the production of reactive oxygen and nitrogen intermediates such as NO and promote strong Th1 responses, while M2 is accompanied by diminished proinflammatory cytokine secretion and shares high expression of scavenger, mannose (CD206) and galactose receptors [5].

Recent work on *T. gondii* polymorphism-associated immune responses show that mouse macrophages infected with type I and III strains are polarized toward an M2 activation state through activation of STAT6, while type II infected macrophages are M1-like cells elicited by activation of NF-κB [3,6]. Type I and type III rhoptry kinase ROP16, inferred as one of virulence determinants, can constitutively activate STAT6, and type II dense granule antigen GRA15 is responsible for strain-specific NF-κB activation [6].

The majority of *T. gondii* strains isolated from humans and animals in North America and Europe have been grouped into three predominant clonal lineages (types I, II and III) that differ genetically by less than 1% [7,8]. Regardless of the genetic background of mice, type I strains are highly virulent with an LD100 = 1, whereas type II or type III strains associate with host genetic background and display lower virulence with an LD50 ≈ 10^2^ and ≈ 10^5^, respectively [9]. Recently there were several studies revealing that a few major clonal lineages of *T. gondii* dominate in different geographical regions. For example, the type 12 lineage is most common in wildlife in North America [10], and the Africa 1 and 3 are among the major types in Africa [11]. Our previous study and other lab research showed that the genotype China 1 (ToxoDB #9) is dominantly prevalent in China [12-15].

The outcome of toxoplasmosis in the mouse model is mainly influenced by the genotype of strain of *T. gondii*[16]. Interestingly, within the genotype China 1, isolates of TgCtwh3 and TgCtwh6 have remarkably different virulence to mice [12], in which TgCtwh3 is highly virulent and cause lethal infection, whereas TgCtwh6 is mildly virulent and able to establish latent infection with cysts in the brain of mice. This virulent difference triggered our interest in understanding the distinction between the two isolates. In the present study, we systematically compared the acute immune response of host, macrophage activation and modulation of the signaling pathway to infection with TgCtwh3 and TgCtwh6 tachyzoites respectively.

## Methods

### Ethics statement

All animal experiments were conducted in strict accordance with the Chinese National Institute of Health Guide for the Care and Use of Laboratory Animals (1998) and approved by the Institutional Review Board of Anhui Medical University Institute of Biomedicine (Permit Number: AMU26-081108). All efforts were made to minimize the animal’s suffering.

### Mice and parasites

Six- to eight-week old BALB/C mice were purchased from the Animal Department of Anhui Medical University and acclimatized for at least 1 week before the experiment. Mice were bred in-house under specific pathogen-free conditions with free access to food and water.

The tachyzoites of mouse-virulent TgCtwh3 isolates or RH strain were harvested from the mouse peritoneal exudates by injection of ice-cold D-Hanks solution on day 3 after infection, and then isolated by centrifugation at 35×g for 5 min to discard contaminating host cells. After the supernatant was centrifuged at 1350×g for 10 min, the parasites were washed once and maintained by serial passages in L929 fibroblast monolayers for further infection experiments *in vitro* and *in vivo*.

The tachyzoites of TgCtwh6 or PRU strain were initially obtained by inoculating brain homogenate containing cysts from infected mice and then cultured with L929 fibroblasts. For initial passages, fibroblast monolayers were detached by scraping, and cells were forced through a 27-gauge needle to release the intracellular parasites. The tachyzoites of TgCtwh6 and PRU were maintained by continual cell passages.

### Cell culture

The mouse macrophage RAW 264.7 cell line was maintained in DMEM (GIBCO) supplemented with 10% heat-inactivated fetal calf serum (FCS, GIBCO), 100 U/ml penicillin, and 0.1 mg/ml streptomycin. Bone marrow-derived macrophages (BMMφs) were generated by culturing bone marrow cells isolated from femurs and tibia of 3–6 months old female Swiss Webster mice in 20% L929 cell-conditioned medium, as previously described [17]. Used as a source of macrophage colony-stimulating factor, L929-conditioned medium was prepared by harvesting supernatants from L929 murine fibroblasts cultured for 6-7d in RPMI 1640, supplemented with antibiotics and FCS as indicated above. All parasite strains and cell lines were routinely checked for mycoplasma contamination, and remained negative throughout the experiments.

### *In vivo* and *in vitro* infection assays

For *in vivo* experiment, 1×10^4^ tachyzoites of TgCtwh3 or TgCtwh6 were inoculated i.p. into mice. After 3 days of infection, mice were euthanized and peritoneal exudate cells (PECs) were harvested by washing the peritoneal cavity with 10 ml of ice-cold D-Hanks solution. PECs were cultured in RPMI 1640, supplemented with 10% FCS, 100 U/ml penicillin, and 0.1 mg/ml streptomycin. Three hours later nonadherent cells were washed off and the remaining adherent macrophages (5×10^6^ cells per well) (termed Wh3-Mφ and Wh6-Mφ, respectively) were cultured in 6-well plates for 12 h.

For *in vitro* infection assays, maintained in 12-well plates (1×10^6^ cells per well), BMMφs and RAW 264.7 cells were pre-stimulated for 24 h with 100 ng/ml recombinant murine IFN-γ (PrimeGEne, China) and 100 ng/ml LPS (Sigma, USA), or with 5 ng/ml rMu IL-13 plus 20 ng/ml IL-4 (PrimeGEne), or left unstimulated, then infected with freshly lysed *T. gondii* tachyzoites at a parasite to host cell ratio of 2:1. Cells were incubated for an additional 24 h.

### Measurement of cytokine levels

After *in vitro* culture for 24 h, supernatants from RH-, PRU-, TgCwh3- or TgCwh6-infected BMMφs were collected and frozen at −80°C. The concentration of IL-12p40 and TNF-α was determined using ELISA kits (Uscnk, USA and MultiSciences, China, respectively) following the manufacturer’s directions.

### Determination of Nitric Oxide (NO) production and arginase activity

Cell culture supernatants from Wh3-Mφ, Wh6-Mφ or normal peritoneal Mφ were collected at 12 h, and supernatants from TgCwh3- or TgCwh6-infected BMMφs or RAW 264.7 cells and uninfected control cells were collected at 24 h after infection. Nitric oxide production was assessed by nitrite accumulation in the culture media using the Griess Reagent System (Promega, USA). Briefly, 50 μl supernatant or standard solution (1.56-100 μm sodium nitrite) were incubated in duplicate with 50 μl of sulfanilamide solution for 5–10 min, then incubated with 0.1% N-1-naphthyldiamine dihydrochloride for 5 min and the OD was measured at 540 nm.

Arginase activity was measured in macrophage lysates by colorimetric method as previously described [18], and defined as the amount of urea produced from total protein of macrophages.

### Flow cytometry

After 3 days of TgCtwh3 or TgCtwh6 infection, PECs were harvested and cultured in RPMI 1640 as described above. Three hours later adherent Wh3-Mφ and Wh6-Mφ were collected, washed twice with PBS containing 3% FCS and labeled with the antibodies of interest at the appropriate dilution for 20 min at 4°C in the dark. The antibodies include FITC-conjugated anti-F4/80, PE-Cy5-conjugated anti-MHCII, PE-conjugated PD-L2 (programmed death ligand, PD-L) (eBioscience, USA), PE-conjugated PD-L1, PE-Cy5-conjugated anti-CD80, APC-conjugated anti-CD86 (BD Pharmingen, USA), Alexa Fluor 647-conjugated anti-CD206 (BioLegend, USA), as well as appropriate isotype control antibodies. Uninfected macrophages were set up in parallel. Cells were washed three times in FACS buffer and fixed in 2% paraformaldehyde before acquisition and analysis (FACSCanto™, BD Biosciences and FlowJo software 7.6).

### RT-PCR analysis

By resuspension in TRIzol reagent (Invitrogen, CA) RNA was prepared from TgCwh3- or TgCwh6-infected BMMφs at 24 h postinfection. Total RNA was extracted and cDNA was synthesized using the RevertAid First Strand cDNA Synthesis Kit (Fermentas, USA) following the manufacturer’s instructions. Relative quantification of the genes of interest was measured by real-time PCR using the SYBR Premix Ex Taq™ (TaKaRa, Dalian, China). The primers (synthesized by Shenggong, Shanghai, China) for amplification of gene fragments are listed in Table 1. Only experiments, in which a distinct single peak was observed with a melting temperature different from that of the no-template control, were analyzed. Real-time PCR of the housekeeping gene GAPDH allowed normalization of the expression of the interested genes and expression relative to non-infected control samples was calculated utilizing the ΔΔC_T_ method. Reactions were carried out in an Applied Biosystems 7500 Fast Real Time PCR System (Applied Biosystems, SA). Experiments were performed in triplicate, and mRNA fold change represents 2^-ΔΔCT^.

**Table 1:** Sequences of oligonucleotide primers used for real-time PCR

**Target**	**Forward primer (5′-3′)**	**Reverse primer (5′-3′)**	**Length(bp)**
TNF-a	GAACTTCGGGGTGATCGGTC	TGTCTTTGAGATCCATGCCG	221
IL-12p40	TGCCAGGAGGATGTCACCT	GGCGGGTCTGGTTTGATGAT	137
iNOS	GTTCTCAGCCCAACAATACAAGA	GTGGACGGGTCGATGTCAC	127
Arginase-1	CTCCAAGCCAAAGTCCTTAGAG	AGGAGCTATCATTAGGGACATC	185
Ym1	AGAAGGGAGTTTCAA ACCTGGT	GTCTTGCTCATGTGTGTAAGTGA	109
GAPDH	CAACTTTGGCATTGTGGAAGG	ACACATTGGGGGTAGGAACAC-3′	224

### Western blotting

BMMφs grown in a 6-well plate were infected with TgCtwh3 and TgCtwh6 parasites respectively (2:1 ratio of parasites to Mφ) for 24 h. After being washed with ice-cold PBS, in the presence of Protease/Phosphatase Inhibitor Cocktail (Cell Signaling, USA) infected cells were lysed by addition of lysis buffer, or nuclear proteins were extracted according to manufacturer’s instructions (NucBuster Protein Extraction Kit, Novagen, German). Then total cell lysates or nuclear proteins were subjected to 8% SDS-PAGE respectively. Proteins were transferred to a nitrocellulose membrane (Millipore Corp, Billerica, MA), which was blocked in TBS/0.1% Tween-20/5% non-fat dry milk and incubated with primary antibodies. The primary antibodies applied included: anti-iNOS and anti-Stat6 (BD Transduction Laboratories, USA), anti-Ym1 (Stemcell Technologies, Canada), anti-arginase 1 (sc-20150, CA), anti-NF-κB p65 (sc-8008), anti-IκBα (sc-847), anti-p-IκBα (sc-8404), anti-Stat6 (pY641, BD PharMingen), anti-*T. gondii* SAG1 (ViroStat, Portland), anti-Stat3 (4904p), anti-pStat3 (9145P) and anti-β-actin antibody (Cell Signaling). After being washed, membranes were incubated with appropriate horseradish peroxidase-conjugated secondary antibodies (Promega, USA). The bound antibodies were visualized by enhanced chemiluminescence using ECL kit (SupreSignal West Pico; Thermo Scientific), and the results were analyzed with ImageJ software.

### Immunofluorescence staining

TgCtwh3 or TgCtWh6 parasites were allowed to invade cells on coverslips and incubated for different time points. Then coverslips were washed twice in PBS and fixed for 20 min at room temperature with 4% paraformaldehyde. After being washed the cells were permeabilized with 0.3% Triton X-100 for 10 min, and blocked in goat serum for 1 h. To determine the nuclear or cytoplasmic NF-κB p65 expression, HeLa cells were simultaneously incubated with anti-NF-κB p65 antibody (ab7970, UK) along with anti-*T. gondii* p30-FITC (6203, ViroStat, Portland) in the dark overnight at 4°C. Coverslips were washed three times in PBS, incubated for 1 h at room temperature with tetramethylrhodamine goat-anti-rabbit IgG (H+ L) (T2769, Invitrogen), and then incubated with Hoechst dye for 5 min for DNA visualization. Finally, coverslips were extensively washed and mounted on a glass slide with anti-fading agent. Cell preparations were examined with an inverted fluorescence microscope (Olympus IX51), and photos were taken using a digital camera (Nikon DS-5M) and NIS-Elements software F3.0.

### Statistical analysis

Data are expressed as mean ± SD. A one-way ANOVA test (Dunnett test) or Student’s *t*-test were used to compare differences among multiple groups or between two unpaired samples, respectively. Differences were considered statistically significant at *P*<0.05.

## Results

### TgCtwh3 and TgCtwh6 strains display different virulence

**Figure 1:**
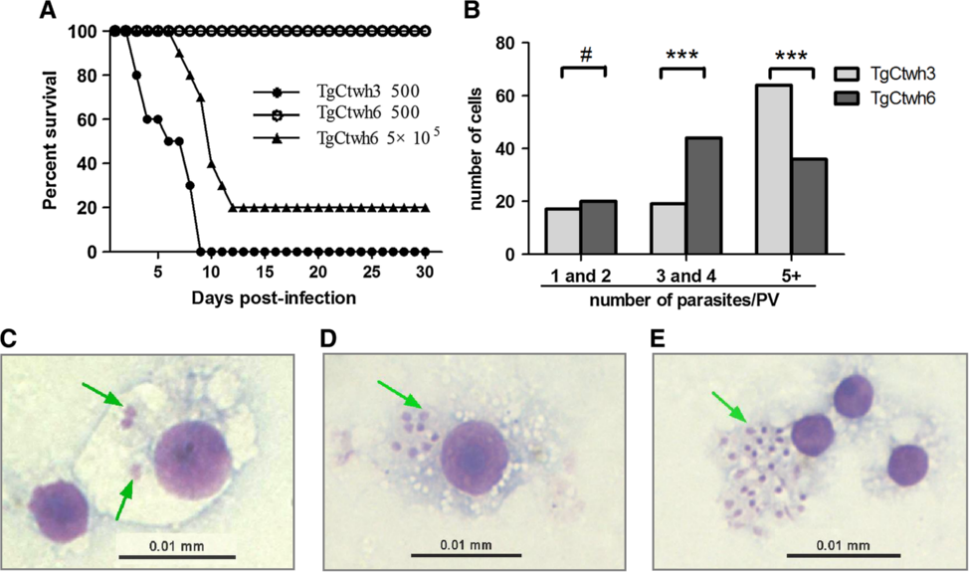
**Survival curve of mice infected with TgCtwh3 or TgCtwh6 and isolates’ growth *in vitro*. (A)** Ten BALB/C mice per group were inoculated i. p. with 500 or 5×10^5^ tachyzoites of TgCtwh3 or TgCtwh6 and monitored for 30 days. **(B)** RAW264.7 cells were infected with TgCtwh3 (gray bars) or TgCtwh6 (dark bars) for 24 h (2:1 ratio of parasites to cells), stained with Wright-Giemsa, and parasites replication was determined by counting the number of parasites from 100 parasitophorous vacuoles by microscopy (Fisher’s exact test, ^***^p<0.001, ^#^p>0.05, TgCtwh3 *vs* TgCtwh6). (**C** and **D**) As in **(B)**, the growth of TgCtwh6 **(C)** and TgCtwh3 **(D)** in RAW246.7 was illustrated. **(E)** Tachyzoites of TgCtwh3 were released from infected RAW264.7 cells. Arrow represents tachyzoites.

Our previous study showed that different from the three clonal lineages of *T. gondii* strains isolated in North America and Europe, the genotype China 1 (ToxoDB#9) is a major lineage prevalent in China with different virulent strains [12]. TgCtwh3 strain leads to fatal infection while mice innoculated with TgCtwh6 can establish latent infection. To evaluate acute virulence, mice were challenged i.p. with low (500 tachyzoites) or high (5×10^5^ tachyzoites) doses of the TgCtwh3 or TgCtwh6 isolates. It was found that mice inoculated with 500 TgCtwh3 tachyzoites uniformly succumbed to infection within 9 days (Figure 1A), in contrast, no death was noted in animals given an equivalent dose of TgCtwh6 tachyzoites during the whole experiment. When infected with 5×10^5^ TgCtwh6 tachyzoites, 20% of the mice survived beyond 30 days and cysts could be found in the brains. Additionally, TgCtwh3 tachyzoites had a higher proliferation (Figure 1B, 1C, 1D) and completely lysed a flask of cultured cells much faster than TgCtwh6 tachyzoites *in vitro* (Figure 1E).

### Distinct cytokine profile of macrophages infected with TgCtwh3 and TgCtwh6 isolates

**Figure 2:**
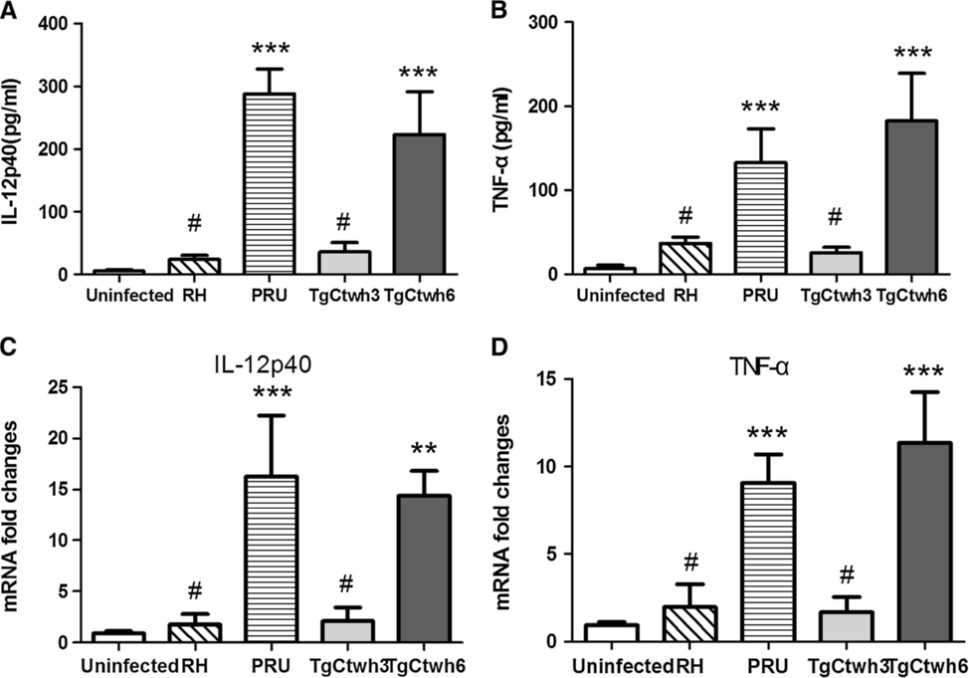
**TgCtwh3 and TgCtwh6 induced different levels of cytokines.** (**A** and **B**) BMMφs were infected with either RH, or PRU, or TgCtwh3 or TgCtwh6 tachyzoites, and 24 hr later supernatants were harvested and cytokine production of IL-12p40 **(A)** and TNF-α **(B)** were determined by ELISA. (**C** and **D**) RNA extracted from RH-, PRU-, TgCtwh3- or TgCtwh6-infected BMMφs, and mRNA was analyzed by real time-PCR using corresponding specific primers. Uninfected Mφ used as negative control was run in parallel. Housekeeping gene GAPDH allowed normalization of the expression of the interested genes. Relative expressions of IL-12p40 **(C)** and TNF-α **(D)** were displayed. Bars represent means ± SD from three independent experiments (^#^p>0.05, ^*^p<0.05, ^**^p<0.01, ^***^p<0.001, parasites infected cells *vs* control).

To examine the production of cytokines that are associated with M1/M2, BMMφs were challenged with tachyzoites of TgCtwh3 and TgCtwh6. The data indicated that TgCtwh6 isolate, rather than TgCtwh3, induced a significant increase of IL-12p40 and TNF-α (Figure 2A, 2B). In concert with protein levels, BMMφs infected with TgCtwh6 also showed elevated mRNA expression of IL-12p40 and TNF-α (Figure 2C, 2D).

### TgCtwh3 and TgCtwh6 down-regulated CD80, CD86 and MHCII molecule expression on macrophages

**Figure 3:**
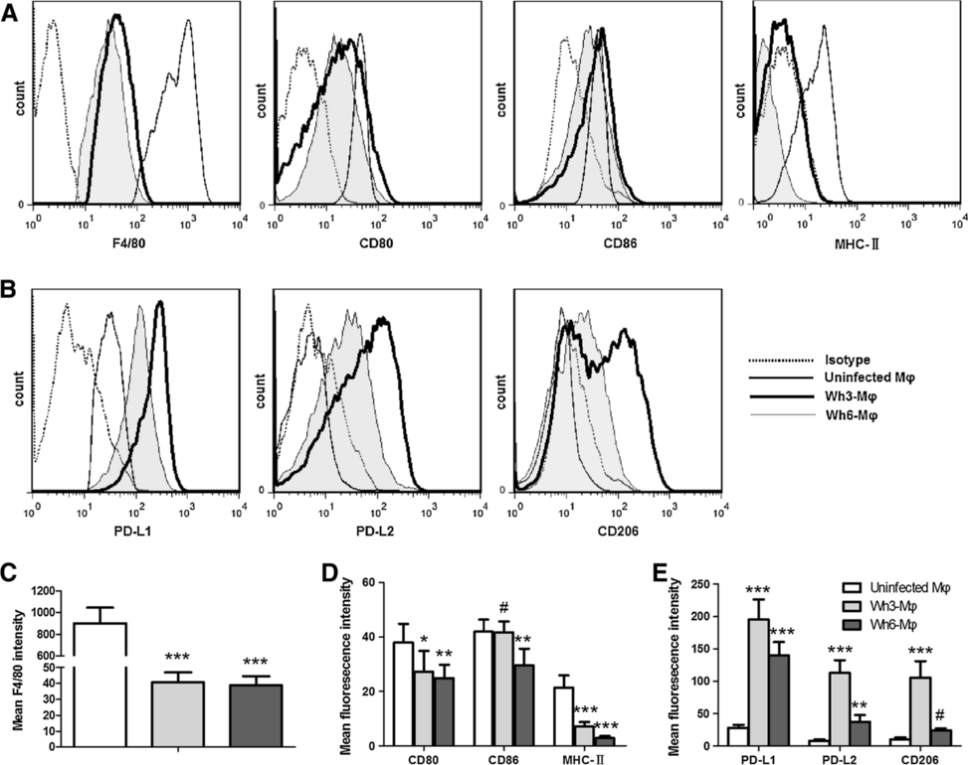
**Cell surface markers in uninfected macrophages, Wh3-Mφ and Wh6-Mφ. (A)** After three days postinfection, mice were euthanized and PECs were harvested. Three hours later adherent peritoneal macrophages were collected and stained for F4/80, CD80, CD86 and MHC class II. **(B)** As in **(A)**, Wh3-Mφ and Wh6-Mφ were stained for F4/80, PD-L1, PD-L2 and CD206. Flow cytometry graphs show histograms of F4/80-gated macrophages (dotted lines, the isotype control; solid lines, uninfected macrophages; heavy lines, Wh3-Mφ; tinted and hairline, Wh6-Mφ). (**C**, **D** and **E**) MFIs of F4/80 **(C)**, CD80, CD86 and MHC class II molecule **(D)**, PD-L1, PD-L2 and CD206 **(E)** on uninfected macrophages, Wh3-Mφ or Wh6-Mφ are displayed (^#^p>0.05, ^*^p<0.05, ^**^p<0.01,^***^p<0.001, Wh3-Mφ or Wh6-Mφ *vs* uninfected Mφ). Data are representative of five independent experiments.

Several studies showed that macrophages infected with *T. gondii* differently regulated the levels of MHC II and co-stimulatory molecules [19-21]. Since the genotype China 1 differs from the archetypal I, II and III strains, the surface expression of CD80, CD86 and MHC class II molecules on peritoneal macrophages of infected mice was quantified by flow cytometry (Figure 3A). The results indicated that both Wh3-Mφ and Wh6-Mφ expressed lower F4/80 than uninfected macrophages, and there was no significant difference in mean fluorescence intensity (MFI) for F4/80 on Wh3-Mφ *vs* Wh6-Mφ (Figure 3C). Additionally, neither Wh3-Mφ nor Wh6-Mφ could up-regulate the expression of CD80, CD86 and MHC class II molecules (Figure 3D).

### Wh3-Mφ up-regulated PD-L2 and CD206 whereas Wh6-Mφ modestly expressed PD-L1

To determine the activation state of macrophages, the expression of CD206, PD-L1 and PD-L2 on peritoneal macrophages of mice was investigated. The results revealed that TgCtwh3 was able to strongly promote PD-L2 expression and Wh3-Mφ was stained positive for CD206, with MFI being 112.78±19.91 and 105.32±25.59, respectively (Figure 3B, 3E). Therefore, Wh3-Mφ displayed features similar to alternative activated macrophages (M2). In contrast, relative to resident peritoneal macrophages, Wh6-Mφ modestly up-regulated the expression of PD-L1 and PD-L2 with MFI being 139.83±20.82 *vs* 27.86±4.88 (p<0.001) and 37.48±10.34 *vs* 5.97±2.96 (p<0.01), respectively (Figure 3B, 3E).

### Wh6-Mφ generated high level of NO whereas Wh3-Mφ up-regulated Ym1 and arginase expression

**Figure 4:**
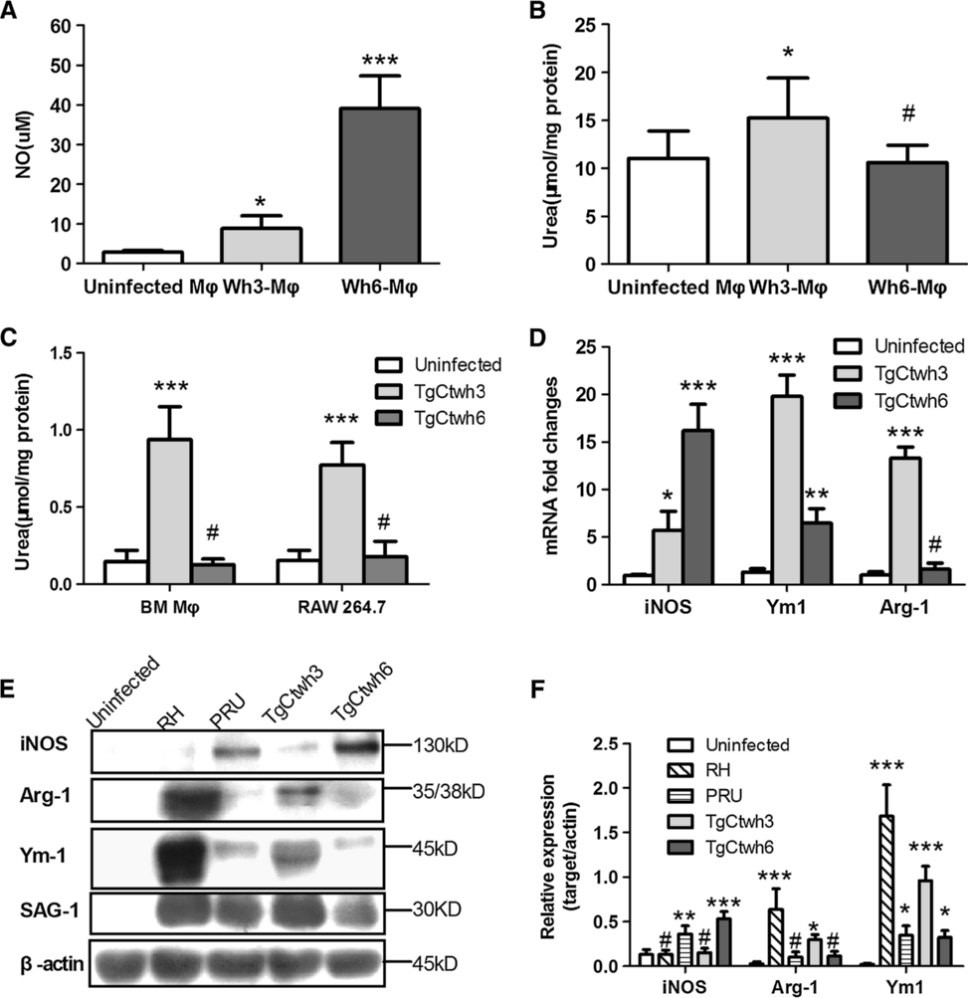
**TgCtwh6 induced high levels of NO whereas Wh3-Mφ had enhanced arginase activity. (A)** On day 3 postinfection with TgCtwh3 or TgCtWh6, mice were euthanized, and adherent peritoneal macrophages from harvested PECs were cultured for 12 h, then supernatants were collected and assayed for NO. **(B)** Arginase activity was measured in Wh3-Mφ or Wh6-Mφ lysates by the conversion of L-arginine to urea. Bars represent means ± SD from five independent experiments (^#^p>0.05, ^*^p<0.05, ^**^p<0.01,^***^p<0.001, Wh3-Mφ or Wh6-Mφ *vs* uninfected Mφ). **(C)** As in **(B)**, infected BMMφs or RAW264.7 cells (2:1 ratio of tachyzoites to cells) were lyzed at 24 h postinfection, and arginase activity was measured (^#^p>0.05, ^***^p<0.001, TgCtwh3 or TgCtwh6 infected cells *vs* uninfected cells). **(D)** RNA was extracted from either TgCtwh3- or TgCtwh6-infected or uninfected BMMφs at 24 h postinfection, and mRNA was analyzed by real time-PCR. Housekeeping gene GAPDH allowed normalization of the expression of the interested genes. Relative expressions of iNOS, Ym1 and Arginase-1 were displayed. Bars represent means ± SD from three independent experiments (^#^p>0.05, ^**^p<0.01, ^***^p<0.001, TgCtwh3- or TgCtwh6- infected cells *vs* uninfected cells). **(E)** Cell lysates collected from BMMφs infected with RH, PRU, TgCtwh3, TgCtwh6 or left uninfected at 24 h postinfection were subjected to Western blotting using antibodies to iNOS, Ym1 and Arginase-1. **(F)** A quantitative analysis of the Western blotting using densitometry (normalized to β-actin) is shown (^#^p>0.05, ^*^p<0.05, ^**^p<0.01, ^***^p<0.001, either RH- or PRU- or TgCtwh3- or TgCtwh6-infected cells *vs* uninfected cells). This experiment was repeated three times with similar results.

To further explore the phenotypic features of activated macrophages infected by TgCtwh3 or TgCtwh6 strains, a variety of *in vitro* and *in vivo* assays were performed on NO and arginase activity, expression of inducible nitric oxide synthase (iNOS), arginase-1 (Arg-1) and Ym1 at transcription and protein levels. As a result, Wh6-Mφ gave rise to robust production of NO which was 14-fold higher than uninfected Mφ (Figure 4A), while TgCtwh3 infection elicited high arginase activity in macrophages as measured by the production of urea (Figure 4B).

To verify the *in vivo* observations, BMMφs or RAW 264.7 cells were infected with either TgCtwh3 or TgCtwh6 isolates for 24 h and the arginase activity was measured. The results indicated that BMMφs or RAW 264.7 cells infected with TgCtwh3, rather than TgCtwh6, had high arginase activity (Figure 4C). To test gene profiles, which are associated with the M1 or M2 phenotypes, the total RNA was extracted from infected BMMφs at 24 h postinfection and the transcriptional response was analyzed by real time PCR (Figure 4D). Consistent with the elevated arginase activity stated above, BMMφs infected with TgCtwh3 displayed a high level of Arg-1 mRNA, 8-fold higher than that with TgCtwh6. The transcription level of Ym1, which encodes a chitinase-like secretory lectin, were also remarkably up-regulated in TgCtwh3 infected BMMφs. In terms of iNOS, TgCtwh6 elicited a marked increase of mRNA compared to uninfected macrophages, which was correlated to the NO production.

Cell lysates collected from BMMφs infected with TgCtwh3 or TgCtwh6 were subjected to Western blotting in order to assay the protein expression of iNOS, Arg-1 and Ym1. Uninfected and RH- (type I), PRU- (type II) infected BMMφs were run in parallel as control. The data showed that RH infection elicited robust Ym1 and high Arg-1 expression but NO was undetectable (Figure 4E, 4F), in contrast to low Ym1 on PRU-infected macrophages. BMMφs infected with TgCtwh3 shared the features of high Arg-1 and Ym1 expression, which were similar to those with RH strain, while TgCtwh6 infection induced strong iNOS expression (Figure 4E, 4F).

### High expression of arginase in M2 is associated with parasite proliferation

**Figure 5:**
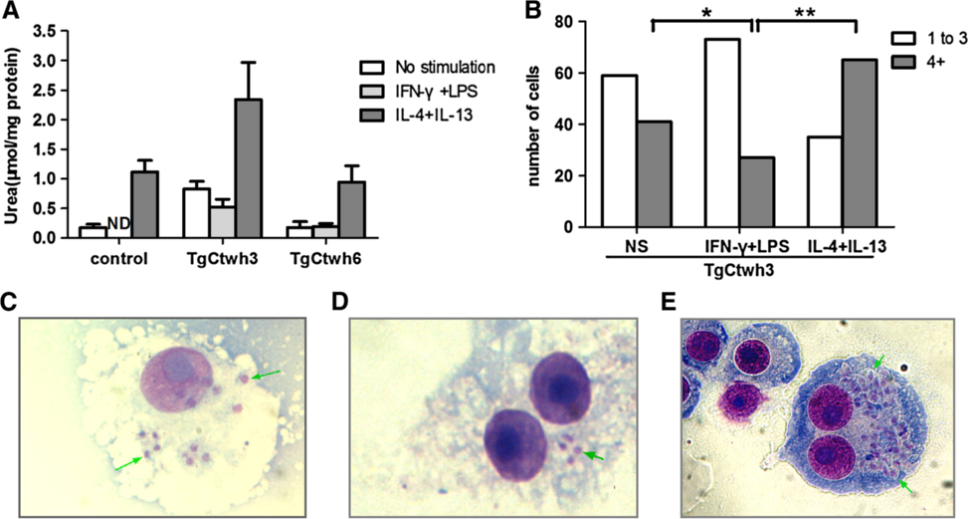
**High arginase activity promoted parasite proliferation. (A)** RAW264.7 cells were either pre-stimulated with 100 ng/ml IFN-γ and 100 ng/ml LPS, or 20 ng/ml IL-4 plus 5 ng/ml IL-13, or left unstimulated for 24 h, then left uninfected (white bars) or infected with TgCtwh3 (grey bars) or TgCtWh6 (black bars) for an additional 24 h (2:1 ratio of tachyzoites to cells), and assayed for arginase activity. **(B)** As in **(A)**, the cells were stained with Wright-Giemsa at 24 h after infection and parasite replication was determined by counting the number of parasites from 100 parasitophorous vacuoles by microscopy. The bars depict the number of cells containing 1–3 parasites (white bars) or ≥4 parasites (dark bars) (Fisher’s exact test was used, ^*^p<0.05, ^**^p<0.01, IFN-γ plus LPS *vs* control or IL-4 plus IL-13). (**C**, **D**, and **E**) The proliferation of TgCtwh3 tachyzoites in RAW264.7 cells: unstimulated **(C)**, pre-stimulated with IFN-γ plus LPS **(D)**, and pre-stimulated with IL-4 and IL-13 **(E)**. Arrow represents tachyzoites. NS: no stimulation.

To evaluate the effects of M1 or M2 on intracellular parasites, RAW 264.7 cells, pretreated either with IL-4 plus IL-13 or IFN-γ plus LPS, were infected with TgCtwh3 and TgCtwh6 respectively, and assayed for arginase activity and parasite growth. In general, arginase activity was enhanced in IL-4 and IL-13-treated cells, especially in those co-infected with TgCtwh3 (Figure 5A). All RAW 264.7 cells, when pre-exposed to IFN-γ and LPS, produced no or less urea. Moreover, the increased proliferation of TgCtwh3 in IL-4 and IL-13-treated cells was also obvious by counting the number of parasites per vacuole (Figure 5B-5E).

### TgCtwh3 induced phospho-STAT6 whereas TgCtWh6 led to activation and nuclear translocation of NF-κB p65

**Figure 6:**
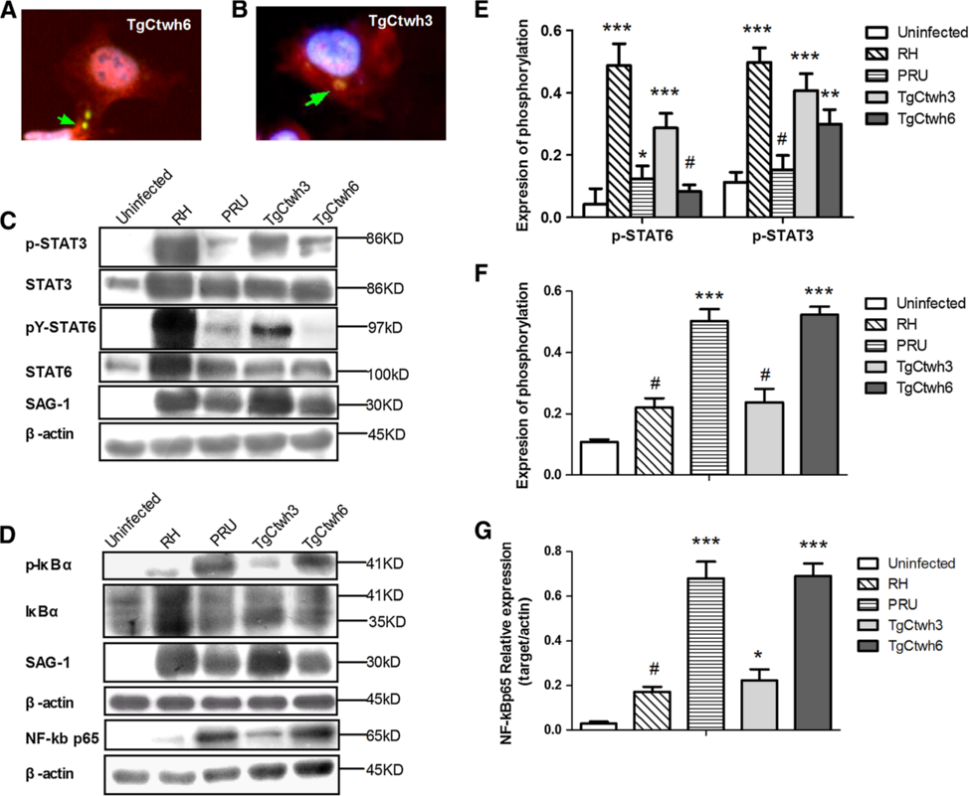
**TgCtwh3 and TgCtWh6 induced activation of macrophages through different signal pathway.** (**A** and **B**) HeLa cells were infected with TgCtwh6 **(A)** or TgCtwh3 **(B)** for 19 hr, fixed, and stained with anti-NF-κB p65 (red), anti-*Toxoplasma gondii* p30-FITC (green), and Hoechst dye (blue). Arrow shows tachyzoites. This experiment has been done five times with similar results. (**C** and **D**) Total lysates were subjected to Western blotting using corresponding antibodies (for STAT6, pY-STAT6, STAT3, p-STAT3, IκBα, p-IκBα and SAG-1) or nuclear lysates (for NF-κB p65 subunit). The lysates were collected from BMMφs infected with either RH or PRU or TgCtwh3 or TgCtwh6 or left uninfected (2:1 ratio of tachyzoites to cells) at 24 h post-infection. (**E** and **F**) The fraction of phosphorylation was displayed. From the total STAT6, STAT3 or IκBα blot, the fraction of phosphorylated STAT6, STAT3 or IκBα was determined by comparing the intensity of the upper band (phosphorylated form) to the total intensity of the lower and upper band. Bars represent means ± SD from three independent experiments (^#^p>0.05, ^*^p<0.05, ^***^p<0.001, either RH- or PRU- or TgCtwh3- or TgCtwh6-infected cells *vs* uninfected cells). **(G)** The nuclear relative expression of NF-κB p65 to nuclear actin using densitometry is also shown. Bars represent means ± SD (^#^p>0.05, ^*^p<0.05, ^***^p<0.001). This experiment was repeated three times with similar results.

To observe nuclear translocation of the NF-κB p65 subunit, HeLa cells were infected with either TgCtwh3 or TgCtwh6 and stained by immunofluorescence (IF). It was found that the cells infected with TgCtwh6 showed high levels of NF-κB p65 in the nuclei, whereas no translocation or only low levels of NF-κB p65 could be noted in the nuclei induced by TgCtwh3 (Figure 6A, 6B).

To further determine the signaling pathway mediated by the isolates of genotype China 1, we preformed Western blotting with total lysates or nuclear lysates from BMMφs infected with either TgCtwh3 or TgCtwh6. Our results showed that, similar to RH, TgCtwh3 induced strong phosphorylation of STAT6 and STAT3, whereas TgCtwh6 triggered less phosphorylated STAT6 and merely moderate activation of p-STAT3 (Figure 6C, 6E). Moreover, results from Western blotting with nuclear lysates demonstrated that, resembling PRU strain, TgCtwh6 tachyzoites were capable of causing the phosphorylation and nuclear translocation of NF-κB p65 subunit (Figure 6D, 6G), which was correlated with the results from IF. By contrast, this response was significantly attenuated in virulent TgCtwh3 tachyzoites.

Since the phosphorylation and degradation of an inhibitory protein IκBα triggers the nuclear translocation of NF-κB transcription factor, the expression of phospho-IκBα was also detected. In parallel with nuclear NF-κB p65, the level of phospho-IκBα in cells infected with TgCtwh6 was obviously higher than in cells infected with TgCtwh3 (Figure 6D, 6F).

## Discussion

Recently we have identified several genotypes of *Toxoplasma* isolates from humans and animals, among them the type China 1 is a dominant clonal lineage prevalent in China [12,14,22]. The present study showed that TgCtwh3 and TgCtWh6 strains dramatically differ in virulence to mice and trigger macrophage-biased activation. Here we found that virulent TgCtwh3 isolate induces the alternatively activated macrophage (M2) phenotype characters by presenting high arginase expression and activity, and STAT6 activation associated with low-level IL-12p40 production. By contrast, less virulent TgCtwh6 isolate activates macrophages similar to classically activated macrophages (M1) with phosphorylation of NF-κB p65 and high secretion of IL-12p40.

Similar to earlier findings that avirulent *T. gondii* strain NTE could down-regulate the expression of MHC class II molecules [20], the present results revealed that both Wh3-Mφ and Wh6-Mφ were completely negative for induction of MHC-II expression (Figure 3A, 3D). Additionally, neither Wh3-Mφ nor Wh6-Mφ could up-regulate CD80 or CD86 expression, which differs from the reports stating that both molecules, or only CD80/CD86 expression, were up-regulated in *Toxoplasma*-infected cells [19,23]. This discrepancy might be the result of different cell types and parasite genotypes used in the studies. It is presumed that down-regulation of CD80 and CD86 along with negative expression of MHC class II molecules might represent a straightforward strategy for China 1 isolates to attenuate the interaction of parasitised antigen presenting cells with specific CD4+ T lymphocytes, as long as the infected cells express insufficient amounts of MHC class II molecules and co-stimulatory signals to stimulate T cell activation.

In addition to expression of CD206, PD-L2 (Figure 3B) and high arginase activity (Figure 4B, 4C), TgCtwh3-infected macrophages possess traits similar to M2, such as up-regulation of Arg-1 and Ym1 at both transcription and protein levels (Figure 4D, 4E). Just as Murry [24] speculated that *T. gondii* I/III strains induce M2 activation bypassing the requirement for exogenous IL-4 and IL-13, it might be unlikely that endogenous IL-4 participates in the skewing of TgCtwh3-infected macrophages, since no IL-4 production was detectable in the supernatants (data not shown). Moreover, it is likely that at an early time of infection, with the activation of M2, the induction of Arg-1 could be exploited by TgCtwh3 to promote its growth as described previously (Figure 5) [25,26]. Early low levels of IL-12 and TNF-α (Figure 2) [27] and migratory dendritic cells and macrophages hijacked *by T. gondii* for parasite dissemination [28] may attribute to later high parasite loads and organ damage [29].

There is plenty of evidence indicating that *T. gondii* actively interferes with host cell signaling during intracellular infection [6,30-32]. The signal transducer and activator of transcription (STAT) signaling pathway as well as NF-κB signaling cascade have emerged as two major targets of exploitation by *T. gondii*[6,33]. Previous data revealed that a single ROP16 polymorphism at position 503 determines the strain difference with respect to STAT3 activation [34], which negatively affects NF-κB signal pathway, leads to suppression of IL-12, and induces differentiation of the infected cells into immune suppressive macrophages [35,36]. Besides, the type I/III ROP16 is capable of directly catalyzing tyrosine phosphorylation of STAT6 [3,33,37,38], and responsible for M2 polarization [3]. Here, we found that although sharing the same 503Leu in ROP16 (additional files show this in more detail [see Additional file 1 and Additional file 2]) and inducing activation of p-STAT3 resemble RH strain, TgCtwh3 and TgCtWh6 isolates trigger notable difference in phosphorylation of STAT6, and promote disparate bias of macrophages. Our results suggest that in addition to ROP16, other potential effector molecules (e.g. ROP18, data not shown) might be involved in the activation of macrophage polarization in China 1 isolates.

On the other hand, macrophages infected with TgCtwh6 give rise to a high production of IL-12p40 and NO, enhanced expression of iNOS on mRNA and protein levels (Figure 4), as well as phospho-IκBα and nuclear translocation of NF-κB p65 (Figure 6). Therefore, it is reasonable to conclude that TgCtwh6 strain activates NF-κB pathway and induces M1-like cells. Thus, TgCtwh6 possesses the features similar to the type II strains (e.g., PRU), such as less virulence and encystation, induction of high IL-12p40 and activation of NF-κB pathway, in spite of alteration of amino acid at 503 of ROP16 and activation of STAT3. Further studies are needed to disclose the polymorphism GRA15 of TgCtwh6 isolate and the polymorphism-associated pathogenesis based on the evidence that type II GRA15 determines M1 polarization [3].

In conclusion, the results indicate that both TgCtwh3 and TgCtWh6 isolates may elicit macrophage-biased responses in different pathways. TgCtwh3 induces activated macrophages resembling M2 through phosphorylation of STAT6, whereas TgCtwh6 activates NF-κB pathway and promotes infected macrophages to skew to a M1 phenotype. The demonstration of different phenotypes among China 1 isolates may be due to the fact that restriction fragment length polymorphism (RFLP) analysis, although valuable for structural population approach, captures only a fraction but not all of the genetic diversity at a given locus. Hence, differences in virulence, cytokine induction and macrophage activation of both isolates make more thorough genetic analysis and analysis of other effectors essential, which may help elucidate the mechanism of the immune response during acute and latent infection with *Toxoplasma* and better understand the influence of strain genotype on human toxoplasmosis.

## Conclusions

The present study demonstrated that macrophages infected with ToxoDB #9 strains of different virulence were polarized toward different activation states through disparate signaling pathway, which may ultimately influence the ability of *Toxoplasma* to establish a chronic infection. To our knowledge, this is the first report of macrophage activation induced by ToxoDB #9 parasites isolated from China. Further studies on other effector molecules may help understand how ToxoDB #9 strains regulate immune responses and provide important clues to disclosure of their essential variance.

## Abbreviations

Mφ: Macrophage; M1: Classically activated macrophage; M2: Alternatively activated macrophage; BMMφ: Bone marrow-derived macrophage; PECs: Peritoneal exudate cells; iNOS: Inducible nitric oxide synthase; PD-L: Programmed death ligand; GRA: Dense granule antigen; NO: Nitric oxide; MFI: Mean fluorescence intensity; ROP: Rhoptry protein; STAT: Signal transducer and activator of transcription.

## Competing interests

The authors declared that they have no competing interests.

## Authors’ contributions

JLS, AMZ, QS, ZRL, and QS conceived and designed the study, and critically revised the manuscript. AMZ, QS, ML and HC performed the experiments and drafted the manuscript. XCX, YW, DYC, LY, and JD participated in the analysis and interpretation of data. YHC and QLL performed the statistical analysis. All authors have read and approved the final manuscript.

## Supplementary Material

Additional file 1 Supplemental methods and the legend of Figure S1. Click here for file

Additional file 2 Sequence alignment of ROP16 alleles. Click here for file
