# Supplementary material for: Comparative studies of macrophage-biased responses in mice to infection with Toxoplasma gondii ToxoDB #9 strains of different virulence isolated from China
Source: Parasit Vectors. 2013 Oct 26;6:308. doi: 10.1186/1756-3305-6-308 (PMC4029513; doi:10.1186/1756-3305-6-308)
Supplement: Additional file 1 — Supplemental methods and the legend of Figure S1. [file 1756-3305-6-308-S1.doc]

**Methods**

**Cloning and sequencing of ROP16 alleles.**

RNA was recovered from freshly egressed tachyzoites by resuspension in TRIzol reagent (Invitrogen, CA). Total RNA was extracted and two μg of RNA were used for the synthesis of cDNA using the RevertAid First Strand cDNA Synthesis Kit (Fermentas, USA). Then the *ROP16* gene was PCR amplified from cDNA with the forward primer: 5’ GAATTCATGAAAGTGACCACGAAAG 3’ and reverse 5’ GGGTACCCCTACAT CCGATGTGAAGAAAGTT 3’. DNA sequences were conducted by Shenggong Technology Services (Shanghai, China). The entire coding sequence of ROP16 of TgCTwh3 and TgCtwh6 was compared with that of GT1 (typeⅠ) and ME49 (typeⅡ) in Toxoplasma database (<http://ToxoDB.org/toxo/>) and aligned using MEGA 5.1 software.

**Figure S1. Sequence alignment of ROP16 alleles.** Amino acid sequence of *ROP16* gene is shown for GT1 (typeⅠ), ME49 (typeⅡ), TgCtwh3 and TgCtwh6, with a dash to indicate identical to GT1 sequence. A red frame displays amino acid residue at 503.
