# Supplementary figures and images for: Comparative studies of macrophage-biased responses in mice to infection with Toxoplasma gondii ToxoDB #9 strains of different virulence isolated from China
Source: Parasit Vectors. 2013 Oct 26;6:308. doi: 10.1186/1756-3305-6-308 (PMC4029513; doi:10.1186/1756-3305-6-308)

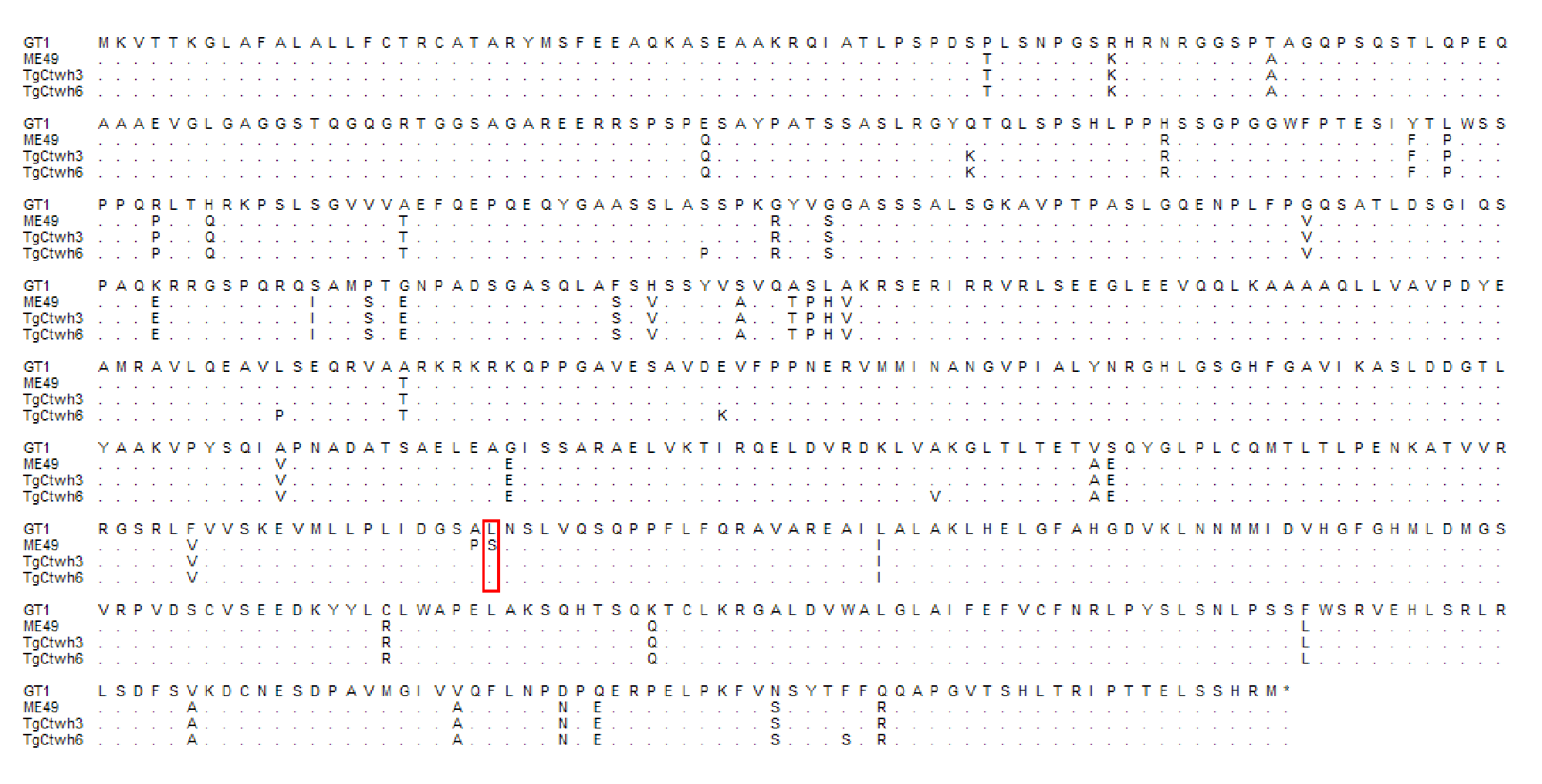

Supplement: Additional file 2 — Sequence alignment of ROP16 alleles. [file 1756-3305-6-308-S2.tiff]
